# Medication Adherence in Patients Undergoing Allogeneic Hematopoietic Stem Cell Transplantation

**DOI:** 10.3390/cancers17152546

**Published:** 2025-08-01

**Authors:** Hermioni L. Amonoo, Emma D. Wolfe, Emma P. Keane, Isabella S. Larizza, Annabella C. Boardman, Brian C. Healy, Lara N. Traeger, Corey Cutler, Stephanie J. Lee, Joseph A. Greer, Areej El-Jawahri

**Affiliations:** 1 Brigham and Women’s Hospital, Boston, MA 02115, USA; edeary@uw.edu (E.D.W.); epkeane@bwh.harvard.edu (E.P.K.); ilarizza@bwh.harvard.edu (I.S.L.); aboardman1@bwh.harvard.edu (A.C.B.); bchealy@mgh.harvard.edu (B.C.H.); 2 Dana-Farber Cancer Institute, Boston, MA 02215, USA; corey_cutler@dfci.harvard.edu; 3 Longwood Medical Area, Harvard Medical School, Boston, MA 02115, USA; jgreer2@mgh.harvard.edu (J.A.G.); ael-jawahri@mgb.org (A.E.-J.); 4 Sylvester Comprehensive Cancer Center, University of Miami, Coral Gables, FL 33136, USA; ltraeger@miami.edu; 5 Fred Hutchinson Cancer Center, University of Washington, Seattle, WA 98195, USA; sjlee@fredhutch.org; 6 Massachusetts General Hospital, Boston, MA 02114, USA

**Keywords:** medication adherence, hematologic malignancy, hematopoietic stem cell transplantation, allogeneic, immunosuppressant, pill counts, patient-reported measures

## Abstract

***Introduction***: Medication adherence is essential for treatment and recovery following hematopoietic stem cell transplantation (HSCT). However, limited data exist on the most effective methods to measure adherence and the factors influencing it in HSCT patients. ***Materials and Methods***: A prospective longitudinal study assessed immunosuppressant medication adherence in 150 patients with hematologic malignancies undergoing allogeneic HSCT. Adherence was assessed using pill counts, immunosuppressant medication levels, patient-reported medication logs, and the Medication Adherence Response Scale-5 (MARS-5) at 30, 100, and 180 days post-HSCT. We evaluated adherence rates, agreement between methods, and sociodemographic and clinical predictors. From patient-reported logs, we calculated dose adherence (comparing reported doses to expected doses) and timing adherence (comparing medication intake within ±3 h of the prescribed time). Kappa analysis assessed agreement among methods. ***Results***: Of 190 eligible patients, 150 (78.9%) enrolled. The mean age was 57.5 years (SD = 13.5); 41.3% (n = 62) were female, 85.3% (n = 128) were non-Hispanic White, and 73.3% (n = 110) were married or living with a partner. Medication adherence varied across the three timepoints and by measurement type: 52–64% (pill counts), 18–24% (medication levels), 96–98% (medication log dose adherence), 83–84% (medication log timing adherence), and 97–98% (MARS−5). There was minimal agreement between measures (Kappa range: 0.008–0.12). ***Conclusions***: Despite the feasibility of leveraging objective and patient-reported measures to assess medication adherence in HSCT patients, there was little agreement between these measures. Patient-reported measures showed high adherence, while objective measures like pill counts and medication levels revealed more modest adherence. The complexity of medication regimens likely contributes to this discrepancy. A rigorous approach to understanding medication adherence in the HSCT population may entail both objective and subjective measures of medication adherence.

## Simple Summary

In patients receiving stem cell transplants from a donor (i.e., allogeneic hematopoietic stem cell transplant), there was a significant difference between how well they reported taking their medications and what the objective data showed. Patients often reported higher medication adherence than what was actually measured. Our findings show that measuring medication adherence is not straightforward, especially because medication regimens can be complicated and change frequently. This emphasizes the need for comprehensive, medication adherence assessments to identify at-risk patients, tailor interventions, and address adherence barriers in clinical practice, ultimately optimizing recovery and treatment outcomes.

## 1. Introduction

Patients undergoing and recovering from allogeneic hematopoietic stem cell transplantation (HSCT) and their caregivers must manage complex medication regimens, including immunosuppressants such as tacrolimus or sirolimus [1]. Medication adherence—the degree to which patients’ medication-taking behavior corresponds to the prescribed regimen from their healthcare provider—is critical to the success of HSCT and the prevention of serious complications, such as graft-versus-host disease (GVHD) [2,3]. However, a significant proportion of patients undergoing HSCT struggle with medication adherence [3]. Some studies show that more than 50% of this population faces challenges with adherence [3,4,5]. Despite this, data are lacking on the optimal approaches for measuring medication adherence and the complex patient factors associated with it in the HSCT population. Research in this area is essential for developing evidence-based interventions to enhance adherence and clinical outcomes.

As in other medical populations with significant comorbidities, multiple factors influence medication adherence among patients undergoing HSCT [6,7,8]. The high number and complexity of medications prescribed for allogeneic HSCT, coupled with co-occurring medical conditions (e.g., GVHD), contribute to challenges with adherence [5]. Additionally, immunosuppressants like tacrolimus have significant intra-patient pharmacokinetic variability, which can be associated with a greater risk of developing GVHD, confounding therapeutic-level testing accuracy [9]. Barriers and facilitators to medication adherence in this population include sociodemographic, clinical, psychological, organizational, and social factors [10,11]. Suboptimal medication adherence in HSCT patients can have several negative short- and long-term consequences, including increased rates of infections, decreased quality of life (QOL), and heightened healthcare utilization, such as hospitalizations and intensive care unit admissions [5]. In the long term, poor medication adherence could also lead to increased mortality [12]. Therefore, a nuanced understanding of the factors associated with suboptimal medication adherence in HSCT survivors is essential for identifying those at highest risk and developing tailored interventions to support these vulnerable patients.

Furthermore, a comprehensive understanding of feasible and effective medication adherence measurement approaches is critical for ongoing efforts to improve adherence in the HSCT population. Although prior studies in other medical populations suggest using a multi-method approach to assess medication adherence [11,12], there are no studies that have incorporated various approaches in the HSCT population. Thus, the goals of this study were to describe immunosuppressant medication adherence using both objective and patient-reported assessment methods, to evaluate agreement across methods, and to characterize potential sociodemographic and clinical factors that may relate to adherence in this population.

## 2. Materials and Methods

### 2.1. Study Procedure

We conducted a prospective longitudinal study of 150 allogeneic HSCT recipients at the Dana-Farber Cancer Institute (DFCI) in Boston, MA, USA, between September 2021 and October 2023. All participants were at least 30 days post-HSCT, a timepoint chosen to assess medication adherence beyond the initial transplant hospitalization. Participants provided written informed consent, and the Dana-Farber/Harvard Cancer Center Institutional Review Board approved the study protocol. Participants were not compensated for study participation.

### 2.2. Participants

Patients were eligible for the study if they were adults (≥18 years of age) with a hematologic malignancy who were at least 30 days post-allogeneic HSCT at DFCI. Eligibility also required that patients be able to speak, read, and complete assessments in English. Exclusion criteria included: (1) pregnancy, and (2) the presence of severe psychiatric disorder (e.g., psychotic disorders) or other comorbid conditions (e.g., dementia) that could preclude successful completion of informed consent or engagement with study procedures, as determined by the primary oncologist.

### 2.3. Sociodemographic and Clinical Data

At baseline (i.e., Day 30 post-HSCT), patients self-reported sociodemographic information, including their race, ethnicity, age, education, income, relationship status, religion, and sex. Data on the number of prescribed medications at various timepoints, along with disease and treatment information, were obtained from the electronic health record (EHR).

### 2.4. Outcome Measures

Participants completed study assessments either during routine in-person clinic visits or electronically via a secure weblink survey. All questionnaires were administered at Day 30 post-HSCT (baseline: (±7 days), Day 100 (±7 days), and Day 180 (±7 days). These follow-up timepoints reflect critical milestones for patients recovering from HSCT.

#### 2.4.1. Objective Measures of Adherence

***Pill Counts***: Immunosuppressants (i.e., tacrolimus or sirolimus) were counted during routine clinic visits at each of the three timepoints: baseline around Day 30 (±7 days), Day 100 (±7 days), and Day 180 (±7 days). Due to limited data for HSCT, we estimated pill count adherence was calculated by determining the number of dosage units taken between two scheduled appointments or clinic visits at each timepoint, based on evidence from solid organ transplantation [13,14,15]. This number was then compared with the total number of units prescribed by the provider, based on EHR data, to calculate the adherence ratio: (Number of dosage units dispensed—number of dosage units remained)/(prescribed number of dosage units per day × number of days between 2 visits) [16,17]. The adherence ratio was then converted to a binary variable, with a ratio between 0.8 and 1.2 indicating adherence and values outside that range indicating nonadherence, as performed in prior studies, with the caveat that these cutoffs may not account for clinically meaningful deviations for the HSCT population [15,18,19,20].

***Immunosuppressant Drug Levels***: All tested drug levels for Tacrolimus or Sirolimus from Day 0 to Day 100 post-HSCT were recorded. We did not assess drug levels at Day 180 because most participants were not on immunosuppressants at that timepoint. Adherence was defined as the proportion of days the patient’s drug levels were within the general therapeutic range [21]. Drug levels were characterized as a binary outcome (adherent versus not-adherent), with adherence defined as having drug levels within the therapeutic range on >80% of days across all timepoints, as has been used in patients undergoing solid organ transplantation, considering the limited data in HSCT [14].

#### 2.4.2. Patient-Reported Measures of Adherence

***Medication Logs and Diaries***: Participants used daily medication logs or diaries for a 2-week period around Days 30, 100, and 180 post-HSCT to record the date, time, dose, and the number of pills taken each time the medication was taken. The medication log diaries allowed us to determine: (a) whether they skipped a dose of their medications; (b) whether they altered their medication dose by specifying if they took more or fewer pills; (c) whether they changed the time they took their medications by specifying how many hours earlier or later than usual they took them, and (d) the reasons for any changes they made to their medications [22]. From this log data, we calculated two measures: “dosage adherence” and “timing adherence.” Dosage adherence was calculated as the number of doses reported as taken compared to the number of doses expected. Timing adherence was defined as medications taken within ±3 h of the prescribed dosing time. A dose taken within the ±3 h window received a full score for that dosing time. A dose taken outside the ±3 h window but within a ±6 h window received a half score, while a missed dose resulted in a score of 0 [23]. Dosage and timing adherence ratios were then computed as binary outcomes (adherent vs. nonadherent) based on adherence ratios between 0.8 and 1.2 being classified as adherent, while ratios outside that range were considered nonadherent, as performed in prior studies [15].

***Medication Adherence Report Scale-5 (MARS-5)***: We also used the 5-item Medication Adherence Report Scale-5 (MARS-5) questionnaire, [24] a widely used self-report tool in medical populations. The MARS-5 assesses a patient’s confidence in managing their medications, including forgetfulness, managing dose changes, discontinuing, skipping, or taking less medication. Similarly to prior work, an A MARS-5 score of ≥23 was classified as adherence, while a score of <23 was considered nonadherent [25,26].

### 2.5. Statistical Analysis

We used STATA 18.0 (StataCorp, College Station, TX, USA) for all analyses. Patients’ characteristics were summarized using descriptive statistics: medians and ranges for continuous variables, and proportions for categorical variables. Descriptive statistics were also used to characterize medication adherence across all timepoints. We conducted unadjusted logistic regression models to assess the relationship between objective and patient-reported adherence measures and sociodemographic and clinical factors. Each adherence measure at baseline and its relationship with participant characteristics were modeled separately. A two-sided *p*-value < 0.05 was considered statistically significant for all analyses.

*Level of Agreement Across Adherence Measures*: We employed kappa analysis to evaluate the level of agreement across adherence measures [27]. Kappa scores are interpreted as follows: ≤0 indicates no agreement, 0.01–0.20 indicates none to slight agreement, 0.21–0.40 indicates fair agreement, 0.41–0.60 indicates moderate agreement, 0.61–0.80 indicates substantial agreement, and 0.81–1.00 indicates almost perfect agreement [28].

## 3. Results

### 3.1. Participant Characteristics

Table 1 presents the characteristics of the study participants. Of the 190 eligible patients, 150 enrolled, resulting in a participation rate of 78.9% (150/190). The average age was 57.5 years (SD = 13.5). Among participants, 41.3% (n = 62) were female, 85.3% (n = 128) identified as non-Hispanic White, 73.3% (n = 110) were married or living with a partner, 66.7% (n = 100) reported being Christian, and 54.0% (n = 81) had attended some college or held a college degree. Most participants (62.7%, n = 94) received reduced-intensity conditioning. By Day 30, 93.3% of participants had not developed acute GVHD.

#### 3.1.1. Objective Measures of Adherence

**Figure 1:**
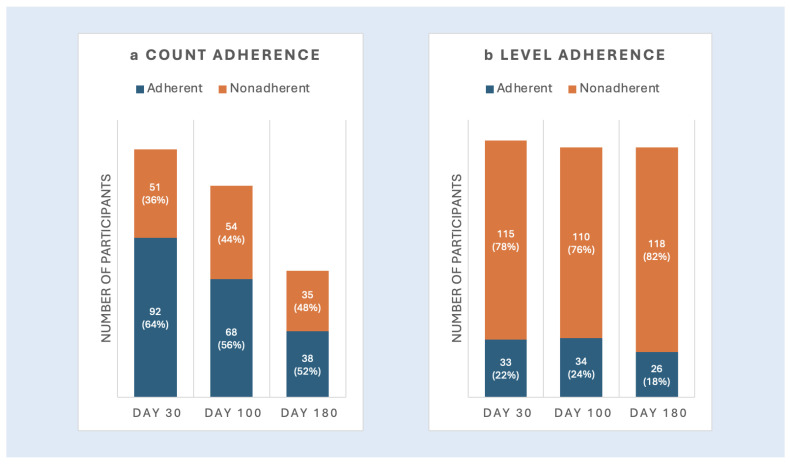
Objective medication adherence across adherence measures and timepoints.

***Medication Adherence* via *Pill Counts***: Figure 1a shows the proportion of participants adherent based on the pill count data. Adherence varied from 64.3% at Day +30, 55.7% at Day +100, and 52.1% by Day +180.

***Medication Adherence* via *Drug Levels***: Figure 1b shows the proportion of participants adherent based on immunosuppressant drug levels. At baseline (Day +30), 22.3% of participants were adherent. By Day +100, 23.6% of participants were adherent.

#### 3.1.2. Patient-Reported Measures of Adherence

**Figure 2:**
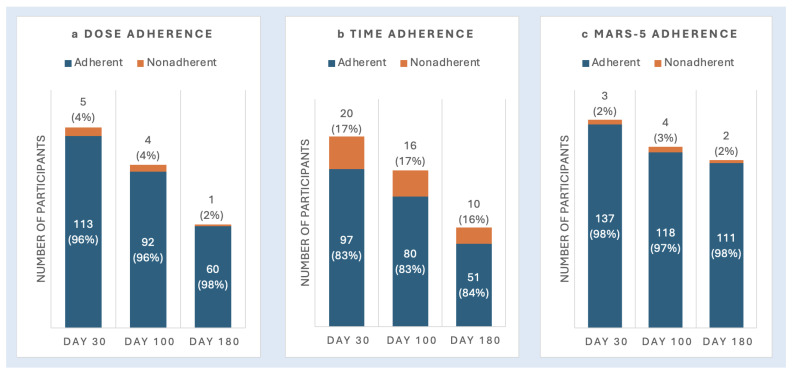
Subjective medication adherence across adherence measures and timepoints.

***Medication Dose* via *Medication Log Data***: Figure 2a displays the proportion of participants adherent to their medication dose according to medication log data. Adherence at baseline, Day +100, and Day +180 was 95.8%, 95.8%, and 98.4%, respectively.

***Time Adherence* via *Medication Log Data***: Figure 2b shows the proportion of participants adherent to the timing of their medications based on log data. At baseline, 82.9% were adherent to timing and at Day +100 (83.3%) and Day +180 (83.6%).

***Medication Adherence* via *MARS-5***: The mean score for all participants who completed the MARS-5 was 24.7 (SD = 0.7) at baseline, 24.7 (SD = 0.9) at Day 100, and 24.6 (SD = 0.8) at Day +180. Figure 2c illustrates the proportion of participants adherent according to MARS-5 scores, with adherence rates of 97.9% at baseline, 96.7% at Day +100, and 98.2% at Day +180.

#### 3.1.3. Level of Agreement Across Measures

Table 2 summarizes the level of agreement across adherence measures at Day +30 post-HSCT, the timepoint when the majority of participants were on immunosuppressants. No agreement was found between the different adherence measures.

Univariate Association between Medication Adherence, Sociodemographic, and Clinical Factors

Supplemental Tables S1–S5 provide details on adherence measures and their univariate and multivariate associations with sociodemographic and clinical factors at Day +30, the timepoint when the majority of participants were on immunosuppressants. No significant associations were observed between these factors and the various adherence measures.

## 4. Discussion

In this longitudinal study of 150 patients with hematologic malignancies undergoing HSCT at a tertiary academic center, we successfully leveraged a multi-method approach to assess medication adherence in this population. While mediation adherence based on patient-reported measures of medication adherence (i.e., medication log data and MARS-5) was high, objective measures such as pill counts revealed only modest adherence. Additionally, there was little to no agreement across the various adherence measures for individual patients, and we did not identify any patient or clinical factors associated with adherence.

This study addresses a significant gap in our understanding of how to assess medication adherence in the HSCT population. Despite the complex and dynamic medication regimens these patients must manage, a multi-method approach to assessing adherence remains feasible in the research context. Previous studies of medication adherence in HSCT patients have not consistently used multi-method approaches, likely due to well-documented challenges (e.g., biases in subjective measures and the labor-intensive nature of objective measures like pill counts) with measuring adherence in medical populations [3,29]. For our study, rigorous training of clinical research coordinators, support from the transplant pharmacy team who works closely with patients, and alignment of adherence measures with routine outpatient clinic visits facilitated implementing the multi-method measurements of adherence in this population. However, further assessment of practical barriers (e.g., staffing, electronic health record integration, and clinician time constraints) to implementing a multi-measure approach to medication measurement will also inform adoption in real-life clinical settings. This study also offers insights into estimating adherence in the HSCT population, drawing on findings from other seriously ill populations [25]. Future research should leverage multi-method measurements in larger cohorts of patients with hematologic conditions undergoing HSCT to further describe adherence in these populations from diverse backgrounds and across care settings.

Medication adherence measured via patient-reported approaches (e.g., medication log data) was higher than adherence measured via objective approaches (e.g., pill counts). There was generally no agreement between these different adherence measures for individual patients. To our knowledge, this is the first study to assess agreement between patient-reported and objective medication adherence measures in the HSCT population. In other medical populations in which adherence has been extensively studied, prior work has shown fair agreement between self-reported and clinician-reported adherence [30]. Several considerations may explain our findings of no agreement between measures. The first consideration is the complexity and fluidity of medications for the HSCT population. For example, a patient may appear adherent based on pill counts but may not have taken the correct dose due to recent changes in their prescription; immunosuppressant doses can change weekly depending on the clinical situation [31]. Additionally, previous qualitative studies have shown that patients often find it challenging to take afternoon medications on time [11]. Hence, it is not surprising that time adherence may not align with pill counts or dose adherence, as a patient may take the correct dose but fail to follow the prescribed schedule accurately. Immunosuppressant drug levels, which are frequently monitored in the HSCT population due to their narrow therapeutic window, guide medication titration based on drug levels [1]. However, common drug–drug interactions (e.g., between antifungal medications and immunosuppressants), individual pharmacokinetics, and external factors unrelated to adherence (e.g., variations in hematocrit levels, body weight [32]) can significantly affect drug levels and making them a less reliable proxy for adherence in this population [33,34]. Further, the low proportion of our cohort with acute-GVHD and high tacrolimus levels correlated with a lower incidence of acute-GVHD, highlighting the potential for misclassification of patients as nonadherent based on our drug level data and our therapeutic range cut-offs [35]. Although self-report measures for this study were administered without patient identifying information, widely used patient-reported outcome measures such as the MARS-5 may have a ceiling effect and can be susceptible to recall and social desirability bias even if data is collected anonymously [36]. While no single measure of adherence is perfect Ref. [16], our findings suggest that data from a given assessment may not necessarily provide a comprehensive view of medication adherence in this population. Clinically, while pill counts are laborious, they may be more feasible and clinically relevant. To inform clinical practice about best approaches to capture medication adherence comprehensively, future studies should explore measurement fidelity of patient-reported measures and pill counts alongside objective measures, such as electronic monitoring devices, especially among patients at high risk for low medication adherence. Additionally, because medication adherence behaviors may change over time, studies with longer-term follow-up beyond six months post-HSCT are needed to assess how medication adherence evolves during HSCT recovery.

We did not identify any participant or clinical factors that put patients at risk for medication non-adherence, although it is well-established that several sociodemographic factors are associated with adherence [12]. While prior studies have reported low medication adherence rates in the HSCT population Ref. [3], adherence based on patient-reported measures was relatively high in our cohort. Several factors could explain this. First, most of our sample reported a higher socioeconomic status characterized by higher education, employment, and income—all of which are associated with better medication adherence. Although we did not specifically examine health literacy, higher educational attainment is associated with increased health literacy Ref. [37], which is commonly associated with medication adherence [38,39]. Hence, our cohort’s higher educational attainment may be linked to increased health literacy and higher rates of medication adherence. Second, a significant portion of our cohort was married or living with a partner. Regardless of marital status, patients report that caregiver support enhances medication management Ref. [11], especially during the acute recovery phase following transplant when patients are newly discharged. Given that most participants in our study reported having spouses or partners, this caregiver support also likely contributed to better medication adherence. Future studies should assess medication adherence in larger, more socioeconomically, racially, and ethnically diverse populations to examine how these factors (e.g., health literacy, social support) influence adherence in varied HSCT samples.

This study has several limitations that warrant discussion. First, the study sample consisted primarily of non-Hispanic White, married individuals with a college education, from an academic medical center. Since sociodemographic factors are associated with medication adherence Ref. [40], our findings may not be generalizable to all populations. Given the small proportion of Black patients in our sample—a group at higher risk for mortality and GVHD [41]—we are unable to assess the role of medication adherence in this relationship. Additionally, low socioeconomic status (SES) is an independent risk factor for poor HSCT outcomes, including GVHD [41]. Medication adherence may be one mechanism through which SES disparities contribute to these outcomes. Hence, future studies should prioritize diverse populations, including those from lower socio-economic and racial and ethnic minority backgrounds. Second, institutional practices related to medication management could impact the resources available for adherence at transplant centers [42]. Therefore, our findings from a single center may reflect unique medication management practices. Future studies should examine medication adherence using larger sample sizes across multiple transplant centers. Multi-center trials are needed to validate our findings and should include comprehensive cost analyses to evaluate the feasibility of implementing a multi-method approach to medication measurement. They are also better suited to assess the association between adherence, survival, and healthcare utilization. Third, our assessment of medication adherence occurred during the first six months post-HSCT, a period when patients typically have frequent follow-up visits with oversight of medication management. Further, patients may be susceptible to social desirability bias (i.e., from contact with study staff about their medications) shown to compromise self-reported medication adherence [43]. As a result, our findings may not apply to patients who are further along in their transplant recovery. Fourth, although our study team thoroughly reviewed all electronic health record data for medication dose changes, our estimates for prescribed dosage units may not be accurate if clinicians did not always document dose adjustments in the health record. Fifth, we did not use electronic medication monitoring devices such as the Medication Event Monitoring Systems, MEMS Caps, because they are burdensome to use and their high cost makes their use prohibitive outside clinical studies [44,45]. Sixth, considering the low kappa values suggesting near-random agreement between measures, our study may have captured fundamentally different aspects of medication adherence in this population. Finally, although data on the impact of intra-patient variability of immunosuppressants on outcomes in allogeneic HSCT recipients is limited, [9] future studies should evaluate its role in this population, considering its clinical relevance as described for solid organ transplant recipients [46].

## 5. Conclusions

In conclusion, medication adherence can be assessed using a multi-method approach that combines objective data with patient-reported measures. However, these measures may reflect different dimensions of medication adherence, often leading to minimal agreement between them. Future research with larger and more diverse populations could provide a better understanding of the patient-related factors influencing medication adherence in this population.

## Figures and Tables

**Table 1:** Baseline Characteristics.

	Total
	N = 150
Age, m (SD)	57.5 (13.5)
Sex, n (%)	
Female	62 (41.3%)
Male	88 (58.7%)
Race, n (%)	
White	138 (92.0%)
Asian	3 (2.0%)
Black	3 (2.0%)
Middle Eastern	1 (0.7%)
Native American	1 (0.7%)
Not listed	4 (2.7%)
Hispanic, n (%)	
No	10 (6.7%)
Yes	128 (85.3%)
Missing	12 (8.0%)
Relationship Status, n (%)	
Single	16 (10.7%)
Relationship/Not living together	8 (5.3%)
Married/Living Together	110 (73.3%)
Separated/Divorced	5 (3.3%)
Widowed/Loss of Partner	4 (2.7%)
Missing	7 (4.7%)
Religion, n (%)	
Agnostic	12 (8.0%)
Atheist	6 (4.0%)
Buddhist	1 (0.7%)
Catholic Christian	63 (42.0%)
Other Christian	37 (24.7%)
Jewish	9 (6.0%)
Muslim	1 (0.7%)
None	18 (12.0%)
Other	3 (2.0%)
Education, n (%)	
<High School Diploma	2 (1.3%)
High School Diploma (GED)	19 (12.7%)
Some College	45 (30.0%)
College	36 (24.0%)
Some Postgraduate/Professional Education	7 (4.7%)
Post-Graduate/Professional	33 (22.0%)
Missing	8 (5.3%)
Employment, n (%)	
Employed	34 (22.7%)
Home-Maker	3 (2.0%)
Disability	61 (40.7%)
Retired	42 (28.0%)
Other	3 (2.0%)
Missing	7 (4.7%)
Cancer Type Combined, n (%)	
Other	36 (24.0%)
Leukemia	83 (55.3%)
Myelodysplastic Syndrome	31 (20.7%)
Transplant Type, n (%)	
Myeloablative Allogeneic	56 (37.3%)
Reduced Intensity Allogeneic	94 (62.7%)
Transplant Setting, n (%)	
Inpatient	138 (92.0%)
Outpatient	12 (8.0%)
Total Body Radiation, n (%)	
No	125 (83.3%)
Yes	25 (16.7%)
Donor Source, n (%)	
Matched	133 (88.7%)
Mismatched	17 (11.3%)
Graft-versus-host Disease, n (%)	
No	140 (93.3%)
Yes	10 (6.7%)
Graft-versus-host Disease Prophylaxis, n (%)	
Cyclophosphamide-Based	33 (22.0%)
Tacrolimus-Based	117 (78.0%)
Combined ecog, n (%)	
2–3	59 (39.3%)
0–1	89 (59.3%)
Missing	2 (1.3%)
Transplant Length of Stay, m (SD)	20.0 (8.7)

**Table 2:** Level of Agreement Across Adherence Measures.

Adherence Measures						Kappa
Pill Count	Dose Adherence		DA Y	DA N	Total	0.025
		PC Y	71	2	73	
		PC N	40	2	42	
		Total	111	4	115	
Pill Count	Time Adherence		TA Y	TA N	Total	−0.097
		PC Y	58	15	73	
		PC N	37	5	42	
		Total	95	20	115	
Pill Count	MARS-5		M Y	M N	Total	−0.044
		PC Y	83	3	86	
		PC N	49	0	49	
		Total	132	3	135	
Pill Count	Drug Level		LA Y	LA N	Total	0.035
		PC Y	8	83	91	
		PC N	2	48	50	
		Total	10	131	141	
Dose Adherence	Time Adherence		TA Y	TA N	Total	0.116
		DA Y	95	18	113	
		DA N	2	2	4	
		Total	97	20	117	
Dose Adherence	MARS-5		M Y	M N	Total	−0.035
		DA Y	104	3	107	
		DA N	5	0	5	
		Total	109	3	112	
Dose Adherence	Drug Levels		LA Y	LA N	Total	0.006
		DA Y	8	105	113	
		DA N	0	5	5	
		Total	8	110	118	
Time Adherence	MARS-5		M Y	M N	Total	0.042
		TA Y	89	2	91	
		TA N	19	1	20	
		Total	108	3	111	
Time Adherence	Drug Level		LA Y	LA N	Total	0.008
		TA Y	7	90	97	
		TA N	1	19	20	
		Total	8	109	117	
MARS-5	Drug Level		LA Y	LA N	Total	0.004
		M Y	10	125	135	
		M N	0	3	3	
		Total	10	128	138	

## Data Availability

For original data, please contact hermioni_amonoo@dfci.harvard.edu.

## Supplementary Materials

The following supporting information can be downloaded at: https://www.mdpi.com/article/10.3390/cancers17152546/s1, Supplementary Table S1: Unadjusted Associations between Count Adherence at Day +30, Sociodemographic, and Clinical Factors, Supplementary Table S2: Unadjusted Associations between Time Adherence at Day +30, Sociodemographic, and Clinical Factors, Supplementary Table S3: Unadjusted Associations between Dose Adherence at Day +30, Sociodemographic, and Clinical Factors, Supplementary Table S4: Unadjusted Associations between Level Adherence at Day +30, Sociodemographic, and Clinical Factors, Supplementary Table S5: Multivariate Associations between Count Adherence at Day +30, Sociodemographic, and Clinical Factors.
