# Supplementary material for: Medication Adherence in Patients Undergoing Allogeneic Hematopoietic Stem Cell Transplantation
Source: Cancers (Basel). 2025 Aug 1;17(15):2546. doi: 10.3390/cancers17152546 (PMC12346867; doi:10.3390/cancers17152546)
Supplement: Supplementary file 1 [file cancers-17-02546-s001.zip › cancers-3728564-supplementary.pdf]

Supplemental Tables

Table S1. Unadjusted Associations between Count Adherence at Day +30, Sociodemographic, and Clinical Factors

Variable	Odds Ratio	95% Confidence Interval		P-Value
**Age**	1.001	0.975	1.027	0.939
**Male Sex**	0.803	0.400	1.610	0.536
**Non-Hispanic White (Yes)**	1.270	0.340	4.749	0.723
**Some College or College Graduate**	0.985	0.365	2.655	0.976
**Some Post-grad, Doctoral, or Other Professional Degree**	1.455	0.471	4.494	0.515
**Unable to Work**	0.471	0.194	1.148	0.098
**Retired**	0.619	0.237	1.620	0.328
**Leukemia**	2.116	0.938	4.772	0.071
**Myelodysplastic Syndrome**	1.889	0.668	5.339	0.230
**Reduced Intensity Allogeneic Transplant**	0.743	0.363	1.521	0.417
**Outpatient Transplant Setting**	6.098	0.758	49.077	0.089
**GVHD (Yes)**	0.529	0.146	1.921	0.333

Table S2. Unadjusted Associations between Time Adherence at Day +30, Sociodemographic, and Clinical Factors

Variable	Odds Ratio	95% Confidence Interval		P-Value
**Age**	0.977	0.937	1.020	0.288
**Male Sex**	1.487	0.566	3.907	0.421
**Some College or College Graduate**	1.112	0.271	4.570	0.883
**Some Post-grad, Doctoral, or Other Professional Degree**	1.000	0.215	4.653	1.000
**Unable to Work**	0.233	0.047	1.149	0.074
**Retired**	0.233	0.046	1.194	0.081
**Leukemia**	1.508	0.453	5.018	0.503
**Myelodysplastic Syndrome**	0.754	0.198	2.877	0.679
**Reduced Intensity Allogeneic Transplant**	0.356	0.111	1.145	0.083
**Outpatient Transplant Setting**	0.308	0.067	1.411	0.129
**GVHD (Yes)**	1.033	0.114	9.348	0.977

Table S3. Unadjusted Associations between Dose Adherence at Day +30, Sociodemographic, and Clinical Factors

Variable	Odds Ratio	95% Confidence Interval		P-Value
**Age**	0.937	0.842	1.043	0.237
**Male Sex**	2.185	0.351	13.594	0.402
**Some College or College Graduate**	3.100	0.492	19.549	0.229
**Unable to Work**	1.433	0.191	10.747	0.726

**Retired**	2.400	0.207	27.781	0.483
**Myelodysplastic Syndrome**	0.639	0.098	4.180	0.640

Table S4. Unadjusted Associations between Level Adherence at Day +30, Sociodemographic, and Clinical Factors

**Variable**	**Odds Ratio**	**95% Confidence Interval**		**P-Value**
**Age**	1.027	0.973	1.084	0.339
**Male Sex**	1.285	0.359	4.595	0.700
**Some College or College Graduate**	0.375	0.058	2.405	0.301
**Some Post-grad, Doctoral, or Other Professional Degree**	1.357	0.240	7.673	0.730
**Unable to Work**	0.246	0.045	1.335	0.104
**Retired**	0.538	0.120	2.419	0.419
**Leukemia**	4.014	0.488	32.983	0.196
**Myelodysplastic Syndrome**	1.100	0.066	18.373	0.947

Table S5. Multivariate Associations between Count Adherence at Day +30, Sociodemographic, and Clinical Factors

**Variable**	**Odds Ratio**	**P-Value**
**Age**	1.003	0.873
**Male Sex**	0.765	0.474
**Non-Hispanic White (Yes)**	0.781	0.743
**Some College or College Graduate**	0.879	0.803
**Some Post-grad, Doctoral, or Other Professional Degree**	1.232	0.725
**Unable to Work**	0.451	0.090
**Retired**	0.769	0.618
**Leukemia**	1.753	0.220
**Myelodysplastic Syndrome**	1.723	0.336
**Reduced Intensity Allogeneic Transplant**	0.656	0.314
**Outpatient Transplant Setting**	7.988	0.056
**GVHD (Yes)**	0.550	0.333

Multivariate regression models controlling for different factors as follows:
Age, sex, race/ethnicity, cancer type, transplant setting, GVHD: controlled for education, marital status, employment status, type of transplant
Education: controlled for relationship status, employment status, and type of transplant
Employment status: controlled for education, relationship, and type of transplant
